# Novel Isoprene-Degrading Proteobacteria From Soil and Leaves Identified by Cultivation and Metagenomics Analysis of Stable Isotope Probing Experiments

**DOI:** 10.3389/fmicb.2019.02700

**Published:** 2019-12-06

**Authors:** Nasmille L. Larke-Mejía, Andrew T. Crombie, Jennifer Pratscher, Terry J. McGenity, J. Colin Murrell

**Affiliations:** ^1^School of Environmental Sciences, University of East Anglia, Norwich, United Kingdom; ^2^School of Biological Sciences, University of East Anglia, Norwich, United Kingdom; ^3^Lyell Center, Heriot-Watt University, Edinburgh, United Kingdom; ^4^School of Life Sciences, University of Essex, Colchester, United Kingdom

**Keywords:** isoprene degradation, isoprene monooxygenase, DNA-SIP, metagenomics, *isoA*

## Abstract

Isoprene is a climate-active gas and one of the most abundant biogenic volatile organic compounds (BVOC) released into the atmosphere. In the terrestrial environment, plants are the primary producers of isoprene, releasing between 500 and 750 million tons per year to protect themselves from environmental stresses such as direct radiation, heat, and reactive oxygen species. While many studies have explored isoprene production, relatively little is known about consumption of isoprene by microbes and the most well-characterized isoprene degrader is a *Rhodococcus* strain isolated from freshwater sediment. In order to identify a wider range of bacterial isoprene-degraders in the environment, DNA stable isotope probing (DNA-SIP) with ^13^C-labeled isoprene was used to identify active isoprene degraders associated with soil in the vicinity of a willow tree. Retrieval by PCR of 16S rRNA genes from the ^13^C-labeled DNA revealed an active isoprene-degrading bacterial community dominated by Proteobacteria, together with a minor portion of Actinobacteria, mainly of the genus *Rhodococcus*. Metagenome sequencing of ^13^C-labeled DNA from SIP experiments enabled analysis of genes encoding key enzymes of isoprene metabolism from novel isoprene degraders. Informed by these DNA-SIP experiments and working with leaves and soil from the vicinity of tree species known to produce high amounts of isoprene, four novel isoprene-degrading strains of the genera *Nocardioides*, *Ramlibacter, Variovorax* and *Sphingopyxis*, along with strains of *Rhodococcus* and *Gordonia*, genera that are known to contain isoprene-degrading strains, were isolated. The use of lower concentrations of isoprene during enrichment experiments has revealed active Gram-negative isoprene-degrading bacteria associated with isoprene-emitting trees. Analysis of isoprene-degradation genes from these new isolates provided a more robust phylogenetic framework for analysis of *isoA*, encoding the α-subunit of the isoprene monooxygenase, a key molecular marker gene for cultivation-independent studies on isoprene degradation in the terrestrial environment.

## Introduction

Out of the many non-methane biogenic volatile organic compounds (BVOC), isoprene (2-methyl-1,3-butadiene) is released to the atmosphere in the greatest amount (approximately 535 Tg y^–1^) (Guenther et al., 2012). Over 90% of the isoprene produced in the biosphere is from terrestrial plants and this can have an important effect on the atmospheric chemistry of the lower troposphere, thus influencing the Earth’s climate (Guenther, 1995). In the atmosphere, isoprene reacts with ozone, hydroxyl and nitrogen oxides, thus depleting ozone and hydroxyl radicals in unpolluted environment (Atkinson and Arey, 2003). In polluted environments, with high nitrogen oxide levels, the result is formation of tropospheric ozone, with consequences for human health and crop yield (Ashworth et al., 2013). These reactions result in a global warming effect, both directly and due to an increased lifetime of methane, for which oxidation by hydroxyl radicals is the primary sink (Folberth et al., 2006). In addition, isoprene secondary oxidation products form particulates, giving rise to haze, smog and cloud condensation nuclei, thus affecting the planetary albedo (Carlton et al., 2009).

In many forest ecosystems, isoprene plays an important role in the production of tropospheric ozone, organic nitrates, organic acids, formaldehyde, carbon monoxide, carbon dioxide, and the generation of secondary organic aerosols (Lelieveld et al., 2008; Surratt et al., 2010). Despite the enormous amount of isoprene emitted to the atmosphere, its short residence time results in low ambient concentrations [<1–5 ppbv over Amazonian, Asian and Greek forests (Hewitt and Street, 1992; Klinger et al., 1998; Harrison et al., 2013)], although below the forest canopy, concentrations can be considerably higher, reaching 40 ppbv (Wiedinmyer et al., 2001; Wiedinmyer et al., 2005).

Approximately 20% of plant species on Earth emit isoprene (Sanadze, 2004) to different extents. Klinger and colleagues categorized isoprene-emitting plants into three categories (high-, low-, and non-emitters) (Klinger et al., 1998). High emitters include *Elaeis guineensis* (oil palm) and *Salix alba* (willow); low emitters include *Mangifera indica* (mango), *Fraxinus* (ash) and *Glycine max* (soybean), and examples of non-isoprene-emitting plants are *Carica papaya* (papaya), *Coffea* (coffee), and *Musa sapientum* (banana) (Hewitt and Street, 1992; Klinger et al., 1998). For isoprene-emitting trees, isoprene emissions typically comprise 1–2% of photosynthetically-assimilated carbon under standard conditions (Harrison et al., 2013). Isoprene is synthesized from dimethylallyl diphosphate by isoprene synthase on the thylakoid membrane of the chloroplast and is released principally through the stomatal pores in leaves (Fall and Monson, 1992). Under stressful conditions, such as high temperatures, high light radiation and drought, the percentage of carbon allocated to isoprene emission from photosynthesis in some trees can increase (Sharkey and Loreto, 1993; Schnitzler et al., 2010), reaching intercellular concentration of isoprene of over 30 ppmv [in fully expanded kudzu leaves (Singsaas et al., 2016)]. Isoprene production may be an important mechanism for plants to stabilize the thylakoid membrane (Velikova et al., 2012). It acts as an antioxidant (Loreto et al., 2001; Vickers et al., 2009) and has recently been proposed to be a signaling molecule, influencing signaling networks, gene expression, and production of certain growth regulators in tobacco and *Arabidopsis* plants genetically-engineered to produce isoprene (Zuo et al., 2019).

The biological consumption of isoprene in soils has been known for some time (Van Ginkel et al., 1987; Fall and Copley, 2000). Different soils have different rates of isoprene uptake (Cleveland et al., 1997) and changes in the isoprene levels to which soils are exposed can produce significant changes in bacterial and fungal communities (Gray et al., 2015). Several isoprene-degrading bacteria have been isolated from soils, marine, estuarine, and freshwater environments. High isoprene-emitting plants and trees may be an abundant source of isoprene for bacteria, and during rainfall bacterial cells on the phyllosphere can migrate to the pedosphere via throughfall and stemflow (Bittar et al., 2018), suggesting that soils beneath isoprene-emitting trees could be a rich source of isoprene degraders. The most-well characterized isoprene-degraders include *Rhodococcus* (van Hylckama Vlieg et al., 2000; Crombie et al., 2015; El Khawand et al., 2016), *Gordonia* and *Mycobacterium* (Acuña Alvarez et al., 2009; Johnston et al., 2017). *Alcaligenes*, *Klebsiella*, *Pseudomonas*, and *Methylobacterium* species have also been retrieved from soil from a waste rubber dumping site (Srivastva et al., 2015; Srivastva et al., 2017), although there are no reports on the mechanisms by which these bacteria degrade isoprene. A putative biochemical pathway for isoprene degradation in *Rhodococcus* sp. AD45 has been proposed (van Hylckama Vlieg et al., 2000) and several of the genes involved are induced by isoprene and transcribed as an operon on a megaplasmid in *Rhodococcus* sp. AD45 (Crombie et al., 2015). All isoprene-degrading bacteria that have been characterized contain at least 10 core *iso* genes corresponding to the isoprene metabolic gene cluster *isoGHIJ, aldH*, and *isoABCDEF* (reviewed by McGenity et al., 2018). The first step in the biodegradation of isoprene is performed by a multicomponent soluble di-iron isoprene monooxygenase (IsoMO) encoded by *isoABCDEF.* The oxygenase component of IsoMO is encoded by *isoA, isoB*, and *isoE* (α_2_β_2_γ_2_); *isoC, isoD*, and *isoF* encode a Rieske-type ferredoxin, coupling protein and a reductase, respectively. Isoprene is oxidized by IsoMO to 1,2-epoxyisoprene. Subsequent metabolic steps involve four remaining genes, *isoGHIJ*, encoding a CoA-transferase (*isoG*), dehydrogenase (*isoH*), glutathione *S*-transferase (*isoI*) and glutathione *S*-transferase-like protein (*isoJ*) (van Hylckama Vlieg et al., 1998, 1999). Epoxyisoprene is conjugated with glutathione by IsoI to produce 1-hydroxyl-2-glutathionyl-2-methyl-3-butene. The dehydrogenase (IsoH) then produces 2-glutathionyl-2-methyl-3-butenoate, the subsequent metabolic fate of which is uncertain.

Linking phylogeny and function of microbes in the environment requires cultivation-independent techniques (Vieites et al., 2009). The gene encoding the putative active site subunit of isoprene monooxygenase, *isoA*, which is highly conserved in extant isoprene degraders, has been used to identify the presence, abundance and diversity of isoprene degraders in different environments (El Khawand et al., 2016; Carrión et al., 2018). Gene probes targeting *isoA* have thus far proved to be a valuable molecular tool with which to assess the abundance and diversity of isoprene-degraders in the environment (Carrión et al., 2018), but as with all such “functional gene probes,” its utility relies on a solid database of *isoA* sequences from isoprene degraders. Therefore, the power of such tools can be improved by capturing a more complete diversity of isoprene degraders, which can be accomplished by methods such as DNA-SIP coupled with metagenomics sequencing (Dumont and Murrell, 2005; Coyotzi et al., 2016). However, just as methanotrophs and other microbes demonstrate niche differentiation in their capacity to consume carbon and energy sources over a range of concentrations, we expect the same to be the case for isoprene-degrading microbes, highlighting the importance of using appropriate enrichment conditions during DNA-SIP experiments.

Using DNA-SIP experiments with ^13^C-labeled isoprene, Crombie et al. (2018) retrieved the near complete genome of an isoprene-degrading *Variovorax* strain from a metagenome constructed using ^13^C-labeled DNA from a SIP experiment with leaves from a poplar tree, suggesting that in the vicinity of isoprene-emitting trees, Gram-negative isoprene degraders may be actively benefiting from this abundant carbon source (Crombie et al., 2018). In order to investigate the diversity of active isoprene-degrading bacteria in soil beneath a willow tree (a tree species known to be a high producer of isoprene) we used DNA-SIP experiments, metagenomics analyses and targeted enrichment experiments, which resulted in the isolation of new Gram-negative and Gram-positive isoprene-degrading bacteria from leaves and soils.

## Materials and Methods

### DNA-Stable Isotope Probing

Soil from the vicinity of a willow tree in Colney, Norfolk, United Kingdom was collected and processed on July 1, 2015 (Supplementary Table S5). Microcosms were set up in triplicate and consisted of 40 ml sterile water and 4 g soil in 2-L sealed bottles with approximately 25 ppmv of either ^12^C or ^13^C-labeled isoprene (El Khawand et al., 2016). The use of this large headspace allowed oxidation of the required amount of isoprene while maintaining a comparatively low concentration. Consumption of isoprene was monitored by gas chromatography (Crombie et al., 2015) and fresh isoprene was replenished when the concentration of the headspace fell below 10 ppmv (Supplementary Figure S1). Incorporation of ^13^C-carbon was estimated, and 10 ml aliquots of the soil suspension were harvested at T_0_, after 6 days (T_1_) and after 7 days (T_2_) (Supplementary Table S1). Aliquots were stored at −20°C until DNA was extracted using the FastSpin DNA soil kit (MP Biomedicals, Santa Ana, CA, US) following the manufacturer’s instructions. DNA (5 μg) was separated into ^13^C-labeled (“heavy”) and ^12^C-unlabeled (“light”) fractions by isopycnic ultracentrifugation as described previously (El Khawand et al., 2016). DNA concentration and density of each fraction was determined with a Qubit^TM^ dsDNA HS Assay kit (Thermo Fisher Scientific, United Kingdom) and an AR200 digital refractometer (Reichert, NY, United States), respectively. Fractions containing heavy and light DNA were identified from plots of DNA abundance vs. fraction density (Supplementary Figures S5, S6) and denaturing gradient gel electrophoresis (Supplementary Figure S7) to visualize and identify fractions containing ^13^C-DNA.

### Identification of Active Isoprene-Degrading Bacteria

Profiles of the bacterial communities in un-enriched (^12^C-DNA) and ^13^C-enriched DNA were examined using bacterial 16S rRNA gene amplicon sequencing [amplified using 27fmod-519mod primers (Grob et al., 2015)] utilizing Roche 454 FLX Titanium instruments and was carried out at MR DNA (Molecular Research LP) (Shallowater, TX, United States) (Dowd et al., 2008; Capone et al., 2011). Samples consisted of an unenriched sample, pooled “light” DNA fractions and pooled “heavy” DNA fractions from ^12^C and ^13^C replicates after 6 days of enrichment, and triplicate “light” and “heavy” DNA fractions from ^12^C and ^13^C enrichments after 7 days of enrichment (Supplementary Figures S5, S6). 16S rRNA gene sequences from these PCR amplicons were processed by stripping the Q25 reads of barcodes and primers. Short sequences (<200 bp), sequences with ambiguous base calls and those with > 6 bp homopolymer runs were removed. Remaining sequences were denoised using a custom pipeline (Dowd et al., 2008), OTUs were clustered at 97% sequence identity, chimeric sequences were removed using Uchime (Edgar et al., 2011) and taxonomy was assigned using BLASTn against the RDPII/NCBI database (v 11.1) (Cole et al., 2014). OTUs with less than 1% relative abundance were grouped together and considered collectively as “others”.

### Shotgun Metagenomics to Investigate the Diversity of Isoprene Metabolic Genes

DNA arising from DNA-SIP experiments with willow soil was analyzed by shotgun metagenomics. DNA sequencing of three samples (un-enriched and pooled enriched heavy ^13^C-DNA isolated from microcosms after 6 and after 7 days of incubation), was carried out by the Centre for Genomic Research at the University of Liverpool by performing paired-end sequencing (2 bp × 150 bp) on an Illumina HiSeq4000 sequencer of libraries generated using a TruSeq Nano kit (Illumina), selecting for 350 bp insert size. Reads were trimmed using trimmomatic (v 0.35) with a minimum window quality score of 20 and a minimum length threshold of 50 bp.

Metaphlan 2.0 (Segata et al., 2012) was used to obtain the taxonomic profile and composition of microbial communities in soil samples enriched with ^13^C-labeled isoprene by comparing the trimmed unassembled metagenome data with clade-specific marker genes from 3,000 reference genomes. The number of raw reads for these samples was 42.6, 44.5 and 52.2 million, and the number of trimmed reads was 41.8, 43.7 and 51.1 million, respectively for the DNA samples sequenced.

Metagenome sequences obtained from ^13^C-labeled DNA after 6 (T_1_) and 7 (T_2_) days of enrichment with ^13^C-isoprene were co-assembled with Megahit (Li et al., 2015), using k-mer lengths from 27 to 127, with other parameters left as default. Metabat 2 version 2.12.1 (Kang et al., 2015) was used for binning (minimum contig size 1,500 bp) with the following command line settings:–verysensitive –p1 90 –p2 85 –pB 20 –minProb 75 –minBinned 20 –minCorr90 –minContig 1500. Quality of bins (completeness, contamination) and taxonomy was assessed using CheckM version 1.0.13 (Parks et al., 2015). Bins containing isoprene metabolic genes were annotated using PROKKA version 1.13 (Seemann, 2014).

Isoprene metabolic genes (*iso* genes) from the metagenome data were located using blast + program (Altschul et al., 1997) by searching for *isoA* sequences in the co-assembled T_1_–T_2_ contigs using, as query, IsoA sequences from known isoprene-degrading sequenced isolates (*Rhodococcus* AD45, *Rhodococcus* PD630, *Gordonia* i37, *Rhodococcus* WS1, *Variovorax* WS9, *Variovorax* WS11, and *Nocardioides* WS12), using an e-value of 1e-4 (tblastn). Contigs containing hits (>70% similarity to *Rhodococcus* sp. AD45 *isoA*) were examined and *isoA* sequences were aligned [ClustalW (Thompson et al., 2002)] with *isoA* sequences from known isoprene-degrading bacteria. Neighbor-joining (Saitou and Nei, 1987) *isoA* trees were constructed using the Maximum Composite Likelihood model in MEGA 7 (Kumar et al., 2016). A bootstrap of 1000 replicates was used to test the robustness of the tree topology (Felsenstein, 2011). *Bona fide isoA* sequences were identified and selected based on e-value, length of sequence and genome context, i.e., adjacent to *isoBCDEF* and the presence in the same contig of other isoprene metabolic genes, such as *isoI*, which encodes a glutathione-transferase essential for the second step in isoprene metabolism (Johnston et al., 2017; McGenity et al., 2018).

### Enrichment and Isolation of Isoprene-Degrading Bacteria From Soils and Leaves

Soil and leaf samples were collected from the vicinity of different isoprene-emitting trees. Soil samples, from beneath willow (*Salix alba*) and oil palm (*Elaeis guineensis*) trees, were collected 10–20 cm from the trunk of the tree, 5–10 cm below the surface. Leaf litter, root residues and stones were removed from samples before use. Leaves were collected from willow and oil palm trees by cutting branches from about 2.5 m above ground (see Supplementary Table S5 for tree location and sampling dates). These tree species were chosen based on their isoprene emission potential which has been reported to be in the region of 37 and 173 μg isoprene produced per g dry weight leaf per hour, respectively (Hewitt and Street, 1992).

Soils were homogenized and enrichments were prepared with 40 ml of sterile water and 4 g soil in 2-L gas-sealed bottles. Leaves from each tree were aseptically cut from their petioles and surface microbes were washed off as described previously (Crombie et al., 2018) with the exception of oil palm when approximately 30 g was used and leaflets were separated from the rachis before washing. Each leaf microcosm consisted of leaf washings suspended in 50 ml of Ewers minimal medium (Dorn et al., 1974). Isoprene was added by injection of isoprene vapor to a concentration of approximately 25 ppmv (withdrawn from the headspace of a 2-ml vial containing a small quantity of liquid isoprene heated for 5 min in a 37°C water bath). Triplicate enrichments of each environmental sample were incubated with shaking at 150 rpm at 25°C in the dark and consumption of isoprene was monitored. Leakage and abiotic depletion of isoprene in incubations was ruled out using no-inoculum control assays (results not shown).

After the initial enrichment, all environmental samples were continuously enriched by subculture in 120 ml glass vials sealed with butyl rubber seals with fresh Ewers minimal medium pH 6.5–7.0 containing 1 μl ml^–1^ of vitamins v10 solution (DSMZ). Isoprene was added and maintained at 25 ppmv in the headspace and enriched samples were transferred into fresh minimal medium every 2–3 weeks. After three transfers, cells were transferred onto solid minimal medium supplemented with isoprene in an airtight container, incubated for 24–48 h at 30°C and then left for 7 to 15 days at room temperature (21°C) to permit the growth of slow-growing bacteria. Selective enrichment of isoprene-degraders was achieved by regular sub-culturing in minimal medium containing isoprene as sole carbon source and subsequent plating onto minimal medium plates with routine checks for purity by microscopy and plating onto rich medium (R2A agar) to check for contaminants. Different phenotypes were selected, colonies transferred back into liquid medium (with 25 ppmv isoprene in the headspace) and optical density of cultures was monitored at 540 nm along with isoprene depletion from the headspace. Isoprene-metabolizing strains were purified, checked for purity by microscopy and re-plating onto R2A agar plates and subsequently maintained on solid minimal medium in the presence of 1% (v/v) isoprene. DNA from isoprene-degrading strains was extracted using the FastSpin DNA soil kit (MP Biomedicals, Santa Ana, CA, United States) according to the manufacturer’s instructions. This DNA was then used as a template for PCR amplification of 16S rRNA and *isoA* genes, cloning and subsequent sequencing (Lane, 1991; Carrión et al., 2018). A list of isolates obtained is given in Supplementary Table S4. Genome sequencing of selected isoprene-degrading bacterial isolates was carried out by MicrobesNG (University of Birmingham, United Kingdom) using Ilumina MiSeq and HiSeq 2500 platforms. Whole genome average amino acid identity (AAI) and *p*-values were determined for novel isoprene-degraders using the Microbial Genomes Atlas (MiGA) webserver, *p*-values representing the probability of genomes not belonging to a specific rank (Rodriguez-R et al., 2018).

## Results

### Identification of Active Isoprene-Degrading Bacteria Using DNA-SIP

**FIGURE 1:**
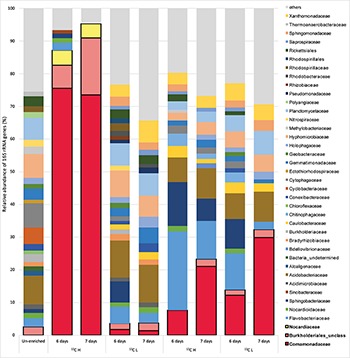
Relative abundance of 16S rRNA genes at the family level in DNA isolated after DNA-SIP enrichments. The relative abundance of bacterial 16S rRNA genes retrieved by PCR from un-enriched (unfractionated) DNA, extracted after sampling, is shown on the left. Subsequent bars show the relative abundance of 16S rRNA genes retrieved from DNA-SIP experiments with isoprene-enriched willow soil samples after 6 and 7 days of enrichment with ^12^C- and ^13^C-labeled isoprene. DNA arising from willow soil samples enriched with ^12^C-isoprene and ^13^C-isoprene are designated as light (L) and heavy (H) fractions respectively (refer to Supplementary Figures S5, S6). Taxonomic affiliation of 16S rRNA genes is reported at the family level. Only 16S rRNA gene sequences with a relative abundance of greater than 1% are shown. 16S rRNA gene sequences with a relative abundance of less than 1% are grouped together as “others.” Families (Comamonadaceae, Burkholderiales unclassified, and Nocardiaceae) identified in these DNA-SIP experiments as putative isoprene-degrading bacteria according to ^13^C-labeling, are highlighted with a black border.

In order to identify active isoprene degraders in the terrestrial environment, DNA-SIP enrichments using fully-labeled ^13^C-isoprene (25 ppmv) were carried out with soil from the vicinity of a willow tree. After 6 and 7 days of incubation, due to rapid degradation of isoprene (T_1_ and T_2_ respectively, Supplementary Figure S1), it was estimated that microcosms had incorporated approximately 25 and 50 μmol g^–1^ of ^13^C-carbon respectively (and in the corresponding ^12^C-isoprene controls, Supplementary Table S1). Comparison of heavy (labeled) and light (unlabeled) DNA fractions from ^13^C-enriched microcosms (and ^12^C-enriched controls) by 16S rRNA gene profiling clearly demonstrated an enrichment of isoprene-degraders after 6 and 7 days (Figure 1).

The 16S rRNA gene sequencing data (consistent across replicates) showed that the bacterial communities in light and heavy DNA fractions in ^12^C-isoprene enrichments were similar to each other, but distinct from the labeled isoprene degraders represented by the heavy DNA retrieved from incubations with ^13^C-labeled isoprene. Comparison of 16S rRNA gene profiles in DNA from un-enriched soil (T_0_ sample) with those from isoprene-enriched samples (6 and 7 days), clearly showed enrichment of members of the Comamonadaceae (Figure 1). These bacteria were present at below 1% relative abundance in unenriched samples but were enriched to around 8–12% relative abundance after 6 days rising to around 21–30% relative abundance after 7 days (Figure 1). Analysis of 16S rRNA gene profiles in ^13^C-labeled (heavy) DNA revealed the major players in isoprene-degradation for the willow soil microcosms were Gram-negative Proteobacteria from the order *Burkholderiales*, which increased from 83 to 91% of the labeled community from T_1_ to T_2_. Of these, the overwhelming majority (91 and 81% respectively) were Comamonadaceae, principally *Ramlibacter* and *Variovorax*, which together accounted for 72 and 59% of the labeled community at T_1_ and T_2_ respectively. Gram-positive Actinomycetales (*Rhodococcus*) also increased in the ^13^C-heavy fraction, but were in a much lower abundance (approximately 4.5% of the labeled community at each time point) (Figure 1 and Supplementary Figure S2).

### Analysis of the Metagenome Derived From ^13^C-DNA Obtained From Isoprene Enrichments

Since the ^13^C-labeled DNA from DNA-SIP soil incubations was enriched in active isoprene-degraders, we examined the diversity of *isoA* genes in this DNA using shotgun metagenomics.

**FIGURE 2:**
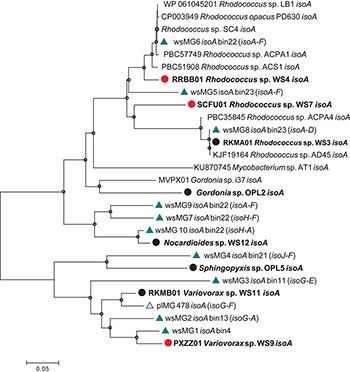
Phylogenetic analysis of *isoA* sequences obtained during this study. Trees were constructed with the neighbor-joining method. The analysis was carried out with 28 complete *isoA* sequences from extant isoprene-degraders, new isoprene-degrading isolates obtained in this study (filled circles, red for isolates from the willow soil DNA-SIP) and 11 complete *isoA* metagenome sequences [green triangles, wsMG (from willow soil SIP-metagenome); blue open triangles, plMG (from poplar leaf metagenome in blue from Crombie et al., 2018)]. Following removal of gaps and missing data, there were 1,428 bp in the alignment. Bin numbers are included following the *isoA*-containing contig identifications. Names of neighboring isoprene metabolic genes in the same contig are included in parentheses. Bootstrap values [1000 replications (Satola et al., 2013)] over 75% are shown as circles in the nodes. The scale bar indicates nucleotide substitutions per site.

Diversity of *isoA* was assessed by extracting *isoA* genes from the assembled metagenome from willow soil enriched with ^13^C-isoprene for 6 and 7 days, using tblastn (Altschul et al., 1990). These *isoA* sequences were then compared with a database of curated *isoA* sequences from isoprene-degrading bacteria (Carrión et al., 2018) together with *isoA* sequences (designated plMG) previously retrieved from metagenome sequencing of ^13^C-DNA retrieved from ^13^C-labeled isoprene enrichments of poplar leaf washings (Crombie et al., 2018). Ten contigs (designated wsMG1 – wsMG10, Figure 2) from the willow soil sequencing of ^13^C-isoprene enrichments contained *isoA* genes. A phylogenetic comparison of these 10 complete *isoA* gene sequences with *isoA* from cultivated isoprene degraders is shown in Figure 2. Phylogenetic analysis clustered several of the willow soil metagenome *isoA* sequences (wsMG6, wsMG5, and wsMG8) with *isoA* sequences from *Rhodococcus* isolates known to degrade isoprene (Crombie et al., 2015, 2017; El Khawand et al., 2016; Johnston et al., 2017). Other *isoA* sequences (wsMG9, wsMG7, and wsM10) were more closely related to *isoA* from isoprene-degrading Actinobacteria such as *Gordonia* and *Mycobacterium* (Acuña Alvarez et al., 2009; Johnston et al., 2017). The remaining four *isoA* sequences (wsMG4, wsMG3, wsMG2, and wsMG1 – see Figure 2) did not cluster with the Actinobacteria, but formed a separate clade most closely related to *isoA* from Proteobacteria, including an *isoA* metagenome-derived contig of *Variovorax*, previous shown to be involved in isoprene degradation (Crombie et al., 2018). The phylogeny of these *isoA* sequences was subsequently revealed by examining the *isoA* genes of new isolates (see below).

### Analysis of Isoprene-Metabolic Gene Clusters Retrieved From DNA-SIP Enrichments

Since the metagenome sequences obtained from DNA-SIP experiments have the potential to contain isoprene metabolic gene clusters from novel isoprene degraders, we examined the contigs containing *isoA* in detail. The *isoA* gene sequences were deemed to be from putative isoprene-degraders as they had a high degree of identity with *isoA* from extant isoprene-degraders (73 to 99% identity at the DNA sequence level to *isoA* from *Rhodococcus* sp. AD45) and they were in contigs containing other clearly identifiable isoprene-specific genes [*isoGHIJ, aldH, isoABCDEF* (Crombie et al., 2015, 2018)].

**FIGURE 3:**
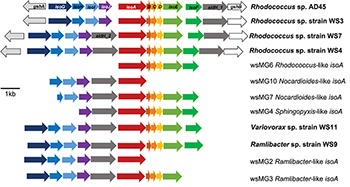
Isoprene metabolic gene clusters. Isoprene metabolic cluster genes identified in isoprene-degrading isolates (bold) and representative *iso* gene-containing contigs obtained from metagenome co-assembly of ^13^C-DNA from isoprene-enriched willow soil (wsMG). The *isoA* gene that encodes for the α-subunit of the monooxygenase is shown in red along with the *isoA* taxonomic affiliation as determined by phylogenetic analysis shown in Figure 2. The % identity of *iso* gene-encoded polypeptides to the corresponding Iso polypeptides of the well-characterized *Rhodococcus* sp. AD45 are shown in Supplementary Table S2.

Five representative *isoA*-containing contigs, with partial isoprene catabolic clusters, were retrieved and compared to complete isoprene catabolic clusters from isoprene-degrading bacteria. Figure 3 compares the isoprene catabolic gene clusters from metagenome-derived *iso* gene clusters retrieved from DNA-SIP experiments with willow soil, with the *iso* genes from the most well-characterized isoprene degrader *Rhodococcus* strain AD45 (van Hylckama Vlieg et al., 2000). Metagenome-derived *iso* clusters from contigs wsMG2, 3, 4, 6, 7, and 10 contained genes *isoABCDEF* that encode the oxygenase, reductase, ferredoxin, and coupling proteins of the isoprene monooxygenase which catalyzes the first step in the isoprene degradation pathway. Some *iso* gene clusters retrieved from metagenome sequences (wsMG2 and wsMG3) also contained *isoGHIJ* which encode the glutathione transferase and dehydrogenase which are required in subsequent steps in the catabolism of isoprene (van Hylckama Vlieg et al., 1999). wsMG4, 6, 7, and 10 also contained one or more of these genes (Figure 3), confirming that the bacteria detected in DNA-SIP experiments are highly likely to be isoprene-degraders, since gene clusters *isoGHIJ, aldH*, and *isoABCDEF* are contiguous on the genomes of these bacteria. The relatedness of the derived polypeptide sequences encoded by the *iso* gene clusters, retrieved through the focused metagenomics approach using DNA-SIP, to the polypeptides IsoGHIJ, IsoABCDEF, and AldH required for isoprene metabolism in isoprene degraders was also examined. The *iso* genes from metagenome contig wsMG6 had the highest degree of relatedness ranging from 77 to 91% AAI (see Supplementary Table S2) to *iso* genes from *Rhodococcus* sp. AD45 and two new isoprene-degrading rhodococci, *Rhodococcus* sp. strains WS4 and WS7, isolated in this study (see below). The taxonomic affiliation of *iso* genes on metagenome-derived clusters wsMG7 and wsMG10 (Figure 3) were subsequently revealed by comparison with the corresponding *isoA* genes from *Nocardioides* strain WS12 isolated from soil (see below). Similarly, isoprene-degrading *Ramlibacter* strain WS9 and *Variovorax* strain WS11 (isolated in this study, see below) could be used to identify the taxonomic affiliation of contigs wsMG2 and wsMG3.

Metagenome assembled genomes (MAGs) were reconstructed by binning contigs from the ^13^C-isoprene enriched metagenomes, and five were selected based on the presence of *isoA-*containing sequences (Figure 2). The quality and taxonomic assignment of *isoA*-containing genome bins was determined, and data are provided in Supplementary Table S3. MAG bin 23 was identified as a *Rhodococcus erythropolis*-like genome (this bin contained 2 *Rhodococcus*-like *isoA* sequences wsMG 5 and wsMG8), and MAG bins 13, 4 and 11 as from the order Burkholderiales (containing wsMG2, wsMG1, and wsMG3 respectively). The 16S rRNA gene amplicon sequencing from ^13^C-labeled heavy DNA showed that the major players in isoprene-degradation were *Ramlibacter*, *Variovorax*, and *Rhodococcus*; the MAGs retrieved are consistent with these results. However, although 16S rRNA gene data did not identify members of the *Sphingomonadales* as abundant (0.27 and 0.55% in ^13^C-labeled heavy DNA after 6 and 7 days of enrichment, respectively), the metagenomics approach identified MAG bin 21 as an isoprene-degrader, containing *iso* genes, from this order.

### Enrichment and Isolation of New Isoprene-Degrading Bacteria

To isolate new isoprene-degrading bacteria, soil samples from beneath the canopy of willow and oil palm trees and epiphyte cells washed off leaves were used in enrichment experiments. Samples were enriched for isoprene-degrading bacteria at an isoprene concentration of approximately 25 ppmv (El Khawand et al., 2016). Isoprene uptake in these microcosms was closely monitored and when isoprene was depleted, it was replenished to 25 ppmv twenty-one times. Seventeen isoprene-degrading isolates were obtained after various rounds of subculture, plating, purity tests and growth in minimal medium with isoprene as a sole carbon and energy source (Supplementary Table S4). All isoprene-degrading isolates grew to an optical density (OD_540_) of greater than 1.0 when grown on 10,000 ppmv isoprene and did not show any growth in the absence of isoprene (or alternative carbon sources). 16S rRNA gene sequencing identified five different genera of isoprene degraders. Of the 13 Gram-positive isolates, 11 were *Rhodococcus* species. These were obtained from willow leaves, soil from beneath a willow tree and from the leaves of an oil palm tree growing in Kew Gardens, London (Supplementary Table S5). This was not unexpected because strains of this genus have previously been isolated from a variety of different environments including soil, freshwater and marine sediments and are known to be important in the biological isoprene cycle (reviewed in Crombie et al., 2018; McGenity et al., 2018). The addition of new *Rhodococcus* strains to our collection of isoprene degraders improved the resolution of the *isoA* phylogenetic tree (Figure 2). Of the remaining two Gram-positive isolates obtained, one strain, *Gordonia* strain OPL2, from oil palm leaves, was most closely related to the isoprene-degrading *Gordonia* strain previously isolated from an estuarine environment (Acuña Alvarez et al., 2009) and described in detail by Johnston et al. (2017). The other Gram-positive isolate was *Nocardioides* sp. strain WS12, isolated from soil taken from beneath a willow tree. No species of this genus have previously been observed to grow on isoprene.

**FIGURE 4:**
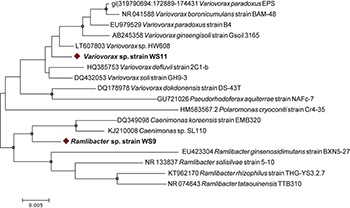
16S rRNA gene phylogenetic tree of representative members of the Comamonadaceae family and isolated strains. Eighteen 16S rRNA gene sequences were included in the Neighbor-joining analysis. Following removal of gaps and missing data, there were 1392 bp in the alignment. Bootstrap values [1000 replications (Satola et al., 2013)] over 75% are shown as circles in the nodes. Strains isolated in this study are shown with a red diamond. The scale bar shows nucleotide substitutions per site.

Since our DNA-SIP experiments described above revealed the presence of active isoprene-degraders in soil which were affiliated to the family Comamonadaceae (Figure 1), we focused on isolation of Gram-negative isoprene degraders from enrichment cultures with samples from willow and oil palm, leading to the isolation of new isoprene utilizers, *Ramlibacter* strain WS9 and *Variovorax* strain WS11 from willow soil samples, and *Sphingopyxis* sp. strain OPL5 isolated from oil palm leaves. Whole-genome analysis using MiGA (Rodriguez-R et al., 2018) shows that our new isolate, *Ramlibacter* sp. strain WS9, isolated from this DNA-SIP experiment, is a member of the Comamonadaceae family. Phylogenetic relationships within this family are not altogether clearly defined (Wen et al., 1999), and await clarification through methods such as phylogenomics. Here, we note that although the 16S rRNA gene of this isolate affiliates it with members of the *Caenimonas* genus (Figure 4), average AAI of whole genome analysis suggests that the strain is from the genus *Ramlibacter*, and the closest species is *Ramlibacter tataouinensis* TTB310 according to the NCBI RefSeq database (68.1% AAI) (Heulin et al., 2003). Data suggest that isolate WS9 was equidistant to both *Ramlibacter* and *Caenimonas* genus, and was considered a *Ramlibacter* for analysis purposes. Comparison of isolate 16S rRNA gene sequences with the OTUs retrieved from the 16S amplicon analysis, revealed that strains represented by our isolates were abundant in the incubations (at 7 days, relative abundance of *Ramlibacter* WS9-like OTUs was 17.7 ± 4.2%; *Variovorax* WS11-like OTUs 6.8 ± 1.3%; *Nocardioides* WS12-like OTUs 0.38 ± 0.3%; *Rhodococcus* WS4-like OTUs 1.6 ± 0.5%; and *Rhodococcus* WS7-like OTUs 1.6 ± 2.2%), see Supplementary Figure S3 for *Ramlibacter*-like OTUs.

The *isoA* genes from each of these new isoprene-degrading strains provided important sequences that improved phylogenetic analysis and identification of *isoA* from Gram-negative bacteria (Figure 2), and also enabled identification of *iso* gene clusters in the metagenomes derived from ^13^C-labeled DNA arising from DNA-SIP experiments (Figure 3). For example, contigs wsMG7 and wsMG10 both contained *isoA* genes with a high degree of identity (88 and 97%, respectively) to the *isoA* gene from *Nocardioides* sp. strain WS12. Contig wsMG4 was also confirmed as containing a *Sphingopyxis*-like *isoA* gene (identity of 83.1% to *isoA* from *Sphingopyxis* sp. strain OPL5). In the case of the newly-isolated isoprene-degrading strains *Ramlibacter* strain WS9 and *Variovorax* strain WS11, comparison of *iso* gene clusters from these isolates and the corresponding genes on contigs wsMG2 and wsMG3 arising from metagenome sequencing of heavy DNA from SIP experiments confirmed that the metagenome sequences were from likely *bona fide* isoprene-degraders (identities of *isoA* were 88 and 83%, respectively).

### Analysis With Newly Isolated Isoprene-Degrading Bacteria

We obtained draft genomes of the novel isoprene-degrading isolates (Table 1). Genome analysis of new *Rhodococcus* strains WS3, WS4, and WS7 revealed that only strain WS3 contained isoprene metabolic gene clusters with the same gene arrangement as found in the well-characterized *Rhodococcus* strain AD45 (Figure 3), which lacks *aldH* in the center of the cluster. The other two rhodococci strains contained *aldH* between *isoABCDEF* and *isoGHIJ*, in common with most known isoprene-degraders. There was also a high degree of identity with these *iso* genes from *Rhodococcus* sp. strains WS3, WS4, and WS7 and the corresponding genes from *Rhodococcus* strain AD45 (99–100 and 77–96% identity at the polypeptide level for WS3 and strains WS4 and WS7, respectively). The *iso* genes from *Ramlibacter* strain WS9 and *Variovorax* strain WS11 encoded polypeptides with a high degree of identity to the corresponding Iso proteins encoded by contigs designated *Variovorax*-like wsMG2 and wsMG3 retrieved in DNA-SIP-derived metagenomes (Figure 3 and Supplementary Table S2). These members of the Comamonadaceae also featured significantly in the ^13^C-DNA retrieved from DNA-SIP experiments with willow soil samples (Supplementary Figure S4).

**TABLE 1:** Data on genomes of isoprene-degrading bacteria isolated in this study together with representative closely related isoprene-degrading and non isoprene-degrading strains.

**Strain**	**Genome size (Mbp)**	**mol% G + C**	**N50**	**Number of contigs**	**Coding sequences**	**tRNAs**	**% Completeness (coverage)^∗^**	**Reference**
***Ramlibacter* sp. strain WS9**	**7.05**	**65.3**	**57140**	**487**	**6614**	**54**	**94.6 (78 x)**	**This study**
*Ramlibacter tataouinensis*TTB310	4.07	70	–	1	3984	47	–	Heulin et al., 2003
***Variovorax* sp. strain WS11**	**8.57**	**67.2**	**96557**	**581**	**8130**	**58**	**95.5 (141 x)**	**This study**
*Variovorax paradoxus* S110	6.75	67.5	5626353	2	6365	67	–	Han et al., 2011
***Gordonia* sp. strain OPL2**	**5.80**	**67.3**	**149633**	**154**	**5372**	**52**	**94.6 (180 x)**	**This study**
***Gordonia* sp. i37**	6.23	66.8	16524	721	5587	53	–	Johnston et al., 2017
***Rhodococcus* sp. strain WS3**	**6.86**	**61.7**		**24**	**6600**	**58**	**94.6 (560 x)**	**This study**
***Rhodococcus* sp. strain WS4**	**12.74**	**66.4**	**40486**	**1261**	**12726**	**65**	**94.6 (28 x)**	**This study**
***Rhodococcus* sp. strain WS7**	**6.63**	**62.4**	**451949**	**183**	**6356**	**65**	**94.6 (64 x)**	**This study**
***Rhodococcus* sp. strain AD45**	6.79	61.7	1521985	9	6522	54	–	Crombie et al., 2015
*Rhodococcus* sp. RHA1	9.70	67.0	7804765	4	9458	64	–	McLeod et al., 2006

*^∗^The characteristics of genomes of isoprene-degrading bacteria (shown in bold) are compared with representative members of their genus. Completeness was determined using the MiGA webserver (Rodriguez-R et al., 2018).*

## Discussion

Although isoprene is one of the most abundant volatile organic compounds released to the atmosphere, there is still relatively little known about the microbes that degrade this climate-active compound. Previous enrichment and isolation studies and DNA-SIP experiments with soil and leaf samples using relatively high concentrations of isoprene (250 ppmv and above) have yielded mainly Actinobacteria of the genera *Rhodococcus, Gordonia*, and *Mycobacterium* (El Khawand et al., 2016; Johnston et al., 2017; Crombie et al., 2018). However, previously there have been hints that the diversity of isoprene degraders in the environment has not been fully realized, for example in DNA-SIP experiments with poplar leaves where an isoprene-degrading *Variovorax* strain was identified (Crombie et al., 2018).

In DNA-SIP and isolation experiments with leaves and soil from the vicinity of known isoprene-emitting trees, we showed, using 25 ppmv as a relatively low concentration of isoprene compared to previous studies (El Khawand et al., 2016; Johnston et al., 2017; Crombie et al., 2018), the presence of a diverse community of isoprene degraders. The Comamonadaceae were the main active members, in contrast to previous DNA-SIP studies (with 150–500 ppmv isoprene), which identified Actinobacteria as the major isoprene degraders. Analysis of *isoA* genes and *iso* gene clusters in metagenomes derived from heavy DNA revealed the presence and relative abundance of new isoprene-degraders and directed the targeted isolation of several new genera, all of which appeared to use the same isoprene-degradation pathway as the well-characterized “workhorse” strain *Rhodococcus* sp. strain AD45. It is also noteworthy that these isoprene-degrading *Rhodococcus* isolates all belong to a genus noted for their relatively large genomes and associated metabolic versatility, particularly with respect to degradation of hydrocarbons (Bröker et al., 2004; McLeod et al., 2006; Kertesz and Kawasaki, 2009; Satola et al., 2013). Experiments of this type can never exclude the possibility that some taxa may not be the primary consumers of labeled substrate but rather become labeled as a result of cross-feeding of labeled metabolic by-products. Here, the relatively short incubation times used in the study, together with isolation of confirmed isoprene degraders from similar taxonomic groups to those identified as labeled, strongly suggests that the labeled sequences belonged to primary isoprene consumers. Furthermore, the low concentrations of isoprene and/or epoxyisoprene may have provided a less toxic environment in which a wider diversity of isoprene degraders could proliferate.

The isolation of new isoprene degraders is important, not only because it provides reference organisms for studying the metabolism of isoprene, but also since it improves the robustness of the “functional gene probe” *isoA* as a diagnostic marker for isoprene degradation in molecular ecology studies (El Khawand et al., 2016; Carrión et al., 2018). Our cultivation-independent investigations of the phyllosphere and of soil from within the vicinity of trees known to emit large amounts of isoprene suggest that microbes play a role in consuming isoprene before it is released to the atmosphere (Fall and Copley, 2000). Isolation of isoprene-degrading *Rhodococcus*, *Nocardioides*, *Ramlibacter*, and *Variovorax* strains from soil near a willow tree were different to the isolates retrieved from oil palm leaves (*Rhodococcus*, *Gordonia*, and *Sphingopyxis* strains). *Rhodococcus* has been isolated from a wide variety of environments, suggesting its ecological flexibility as an isoprene degrader, but our preliminary data suggest that different isoprene-emitting trees have different isoprene degraders in their microbiomes and this warrants further study in the future.

Oil palm are one of the highest emitters of isoprene (Hewitt and Street, 1992) and there are now serious concerns about the huge amounts of isoprene emitted by large oil palm plantations, and the consequent reactions with nitrogen oxides which affect air quality (Hewitt et al., 2009; Sharkey and Monson, 2014). The caveat here however is that the oil palm tree from which we obtained leaves was from the Palm House at Kew Gardens, London, rather than from a tropical plantation, but clearly the phyllosphere of oil palm trees is an important environment for future study of isoprene degraders and to investigate their impact on the biogeochemical cycling of isoprene in soil and leaves in native oil palm plantations. The phyllosphere of trees is a rich source of microbes that can benefit from volatile organic compounds (e.g., methanol) released by plants (Vorholt, 2012) and this may well be the case for isoprene. Rainfall may wash these microbes onto the soil beneath (Bittar et al., 2018) and so the environments below the forest canopy may also be a rich source of isoprene-degraders.

## Conclusion

In this study, DNA-SIP experiments with willow soil enriched with lower concentrations of ^13^C-labeled isoprene than previously, revealed novel and active isoprene-degrading Proteobacteria. 16S rRNA gene sequencing and metagenome analyses confirmed the dominance of Gram-negative isoprene-degrading members of the Comamonadaceae family, in contrast to previous studies which identified Actinobacteria (including *Rhodococcus*) as the major isoprene-degrading taxa. The data suggest that the leaves of isoprene-emitting trees and the soils beneath harbor distinct isoprene-degrading bacterial communities, and significantly increases our knowledge of the diversity and abundance of tree-associated isoprene-degrading bacteria. Cultivation-independent and isolation techniques have extended the isoprene (*iso*) metabolic gene database and novel isoprene-degrading isolates *Ramlibacter*, *Variovorax*, *Nocardioides*, *Sphingopyxis*, and *Gordonia* will provide abundant material for future laboratory studies to understand the molecular mechanisms by which isoprene degradation is regulated in bacteria.

## Data Availability Statement

The datasets generated for this study can be found in the GenBank repository (https://www.ncbi.nlm.nih.gov/bioproject/PRJNA272922).

## Author Contributions

NLL-M, ATC, TJM, and JCM planned the experiments. NLL-M carried out the experimental work. NLL-M, ATC, and JCM analyzed the results. NLL-M and JP conducted the metagenome analysis. NLL-M, ATC, and JCM wrote the manuscript. All authors read, reviewed, and approved the final manuscript.

## Conflict of Interest

The authors declare that the research was conducted in the absence of any commercial or financial relationships that could be construed as a potential conflict of interest.
