# Supplementary material for: Novel Isoprene-Degrading Proteobacteria From Soil and Leaves Identified by Cultivation and Metagenomics Analysis of Stable Isotope Probing Experiments
Source: Front Microbiol. 2019 Dec 6;10:2700. doi: 10.3389/fmicb.2019.02700 (PMC6908491; doi:10.3389/fmicb.2019.02700)
Supplement: Supplementary file 1 [file Presentation_1.pdf]

Table S1. Incorporation of ^{13}C-labelled carbon during DNA-SIP experiments with willow soil

Length of Enrichment	^{13}C incorporation (μmol g^{-1} *)	Time (h)
Un-enriched (T_0)	0	0
Day 6 (T_1)	22.3	138
Day 7 (T_2)	47.3	172.5

* Consumption of isoprene in DNA-SIP microcosms was monitored by gas chromatography. Incorporation of carbon was estimated assuming that 50% isoprene consumed was assimilated.

Table S2. Identity of Iso polypeptides encoded by *iso* gene clusters when compared to the corresponding Iso polypeptides from *Rhodococcus* sp. strain AD45

Isoprene-degrading isolates are in bold. wsMG designations indicate *iso* gene-containing contigs retrieved from willow soil metagenomes. Percentage identities (% amino acid identity) are with respect to the Iso polypeptides of the most well-characterised isoprene-degrader *Rhodococcus* sp. AD45 (Crombie et al., 2015).

100-90	89-80	79-70	69-60	59-50	49-35

**Isolate/contig**<br>***Rhodococcus* sp. AD45**	**IsoG**	**IsoH**	**IsoI**	**IsoJ**	**-**	**IsoA**	**IsoB**	**IsoC**	**IsoD**	**IsoE**	**IsoF**	**AldH_1**
***Rhodococcus* sp. strain WS3**	100	100	99	100	-	100	100	100	100	100	100	100
***Rhodococcus* sp. strain WS7**	90	88	86	77	**AldH_2**	91	77	82	94	83	77	74
***Rhodococcus* sp. strain WS4**	91	88	86	80	94	90	84	87	96	83	81	77
wsMG6 *Rhodococcus*-like *isoA*	-	-	-	-	-	91	84	86	95	84	81	-
wsMG10 *Nocardioides*-like *isoA*	-	73	66	66	63	84	-	-	-	-	-	-
wsMG7 *Nocardioides*-like *isoA*	-	-	73	70	67	85	56	66	68	66	53	-
wsMG4 *Sphingopyxis*-like *isoA*	-	-		57	51	72	44	50	56	51	42	-
***Variovorax* sp. strain WS11**	60	48	48	55	52	73	38	40	58	53	41	-
***Ramlibacter* sp. strain WS9**	61	47	47	55	50	71	50	55	55	53	43	-
wsMG2 *Ramlibacter*-like *isoA*	63	49	49	55	50	71	-	-	-	-	-	-
wsMG3 *Ramlibacter*-like *isoA*	62	49	49	52	53	70	49	55	54	53	-	-

Percentages are rounded to the nearest whole number, except that all values in the range 99 – 100 are shown as 99.

Table S3. Quality assessment and taxonomic assignment of genomic bins reconstructed from metagenome sequence data obtained with ^{13}C-labelled heavy DNA from DNA-SIP experiments.

Data provided for bins with *isoA*-containing contigs, having more than 10% completeness, and less than 10% contamination, as determined using MiGA (Rodriguez-R et al., 2018). NA, Not assigned.

	**Taxonomy**							
**Bin ID**	**Order**	**Family (Species)**	**Completeness (%)**	**Contamination (%)**	**Strain heterogeneity**	**Size (Mbp)**	**% mol GC**	**wsMG *isoA*-containing contig**
23	*Actinomycetales*	*Nocardiaceae* (*R. erythropolis)*	73	2	40	5.99	61.6	5 and 8
21	*Sphingomonadales*	NA	49	0	0	1.94	62.2	4
13	*Burkholderiales*	NA	38	9	12.12	2.79	68.9	2
4	*Burkholderiales*	*Comamonadaceae*	30	2	0	4.30	65.3	1
11	*Burkholderiales*	*Comamonadaceae*	11	0	0	7.16	60.8	3

Table S4. Isoprene-degrading bacteria isolated during this study.
Isolates were obtained from enrichments using soil and leaves from isoprene-emitting trees. Isolates obtained from the DNA-SIP experiment with soil from the vicinity of a willow tree are indicated in bold. Taxonomic classification and p-value for isolates sequenced in this study were determined with the MiGA webserver (Rodriguez-R et al., 2018). Refer to Supplementary Table 5 for sampling dates and exact location of the isoprene-emitting trees. WS: willow soil, WL: willow leaves, OPL: oil palm leaves.

Isolate	Environment	Taxonomic classification (p-value)*
Rhodococcus sp. strain WS1	WS (Colney, UK)	
Rhodococcus sp. strain WS2	WS (Colney, UK)	
Rhodococcus sp. strain WS3	WS (Colney, UK)	*Rhodococcus* sp.
***Rhodococcus* sp. strain WS4**	**WS SIP (Colney, UK)**	*Rhodococcus opacus* (0.05)
***Rhodococcus* sp. strain WS5**	**WS SIP (Colney, UK)**	
***Rhodococcus* sp. strain WS6**	**WS SIP (Colney, UK)**	
***Rhodococcus* sp. strain WS7**	**WS SIP (Colney, UK)**	*Rhodococcus erythropolis* (0.001)
***Rhodococcus* sp. strain WS8**	**WS SIP (Colney, UK)**	
***Ramlibacter* sp. strain WS9**	**WS SIP (Colney, UK)**	*Ramlibacter* sp.
Rhodococcus sp. strain WS10	WS (Colney, UK)	
Variovorax sp. strain WS11	WS (University of East Anglia, UK)	*Variovorax* sp.
Nocardioides sp. strain WS12	WS (University of East Anglia, UK)	
Variovorax sp. strain WS13	WS (University of East Anglia, UK)	
Rhodococcus sp. strain WL1	WL (University of East Anglia, UK)	
Rhodococcus sp. strain OPL1	OPL (London, UK)	
Gordonia sp. strain OPL2	OPL (London, UK)	*Gordonia* sp.
Sphingopyxis sp. strain OPL5	OPL (London, UK)	

*p-values indicate the probability of incorrect taxonomic ranking at that level (Rodriguez-R et al., 2018).

Table S5. Location of environmental samples used during this study.
The table includes the dates at which soil and leaf samples were taken and a short description of the experiments carried out with each. The sample used for DNA-SIP enrichments is shown in bold.

Sample	Location	Coordinates	Dates	Description
**Willow tree soil**	**Colney sport fields (Colney, UK)**	**52°37'17.2"N 1°13'46.3"E**	April 2015 and **July 2015**	**Used for enrichment and isolation of isoprene degraders, and for DNA-SIP experiments**
Willow tree soil	UEA broad (Norwich, UK)	52°37'06.6"N 1°14'09.9"E	June 2016	Used for enrichment and targeted isolation of isoprene degraders
Willow tree leaves	UEA broad (Norwich, UK)	52°37'06.6"N 1°14'09.9"E	September 2015 and October 2016	Used for enrichment and isolation of epiphytic isoprene-degraders
Oil palm tree soil	Kew Gardens Palm House (London, UK)	51°28'43.6"N 0°17'33.1"W	November 2016 and February 2017	Used for enrichment and isolation of isoprene degraders
Oil palm tree leaves	Kew Gardens Palm House (London, UK)	51°28'43.6"N 0°17'33.1"W	November 2016 and February 2017	Used for enrichment and isolation of epiphytic isoprene-degraders

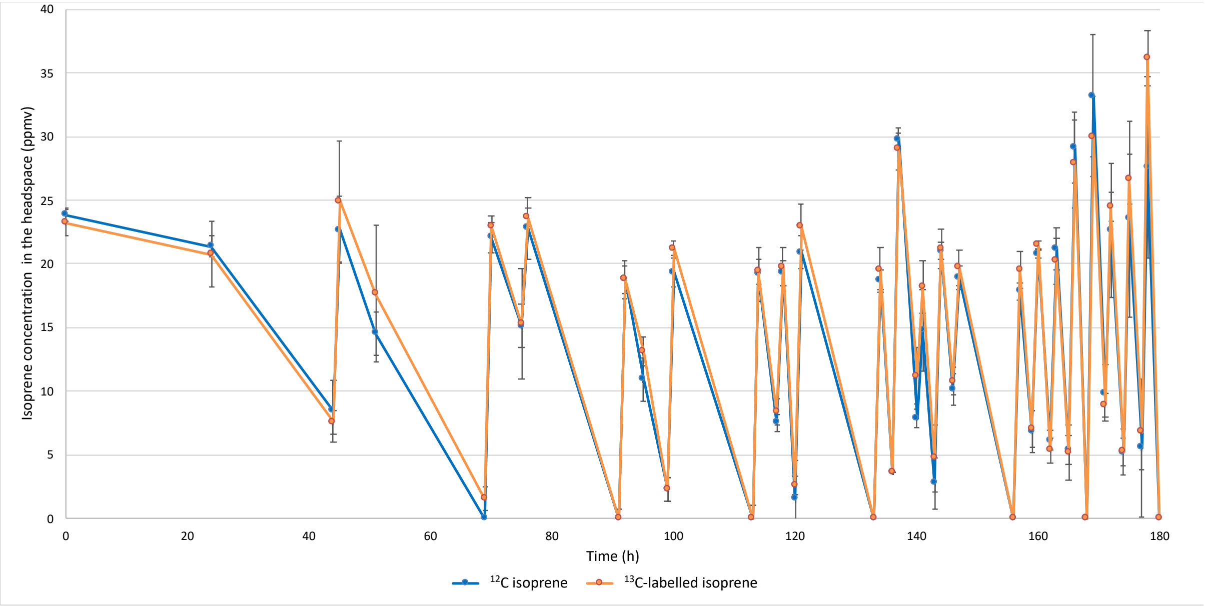

Figure S1. Isoprene consumption in microcosms used in DNA-SIP experiments with willow soil.

Microcosms were enriched with ^{12}C- or ^{13}C-labelled isoprene. Data points show the mean of triplicates and error bars show the standard deviation.

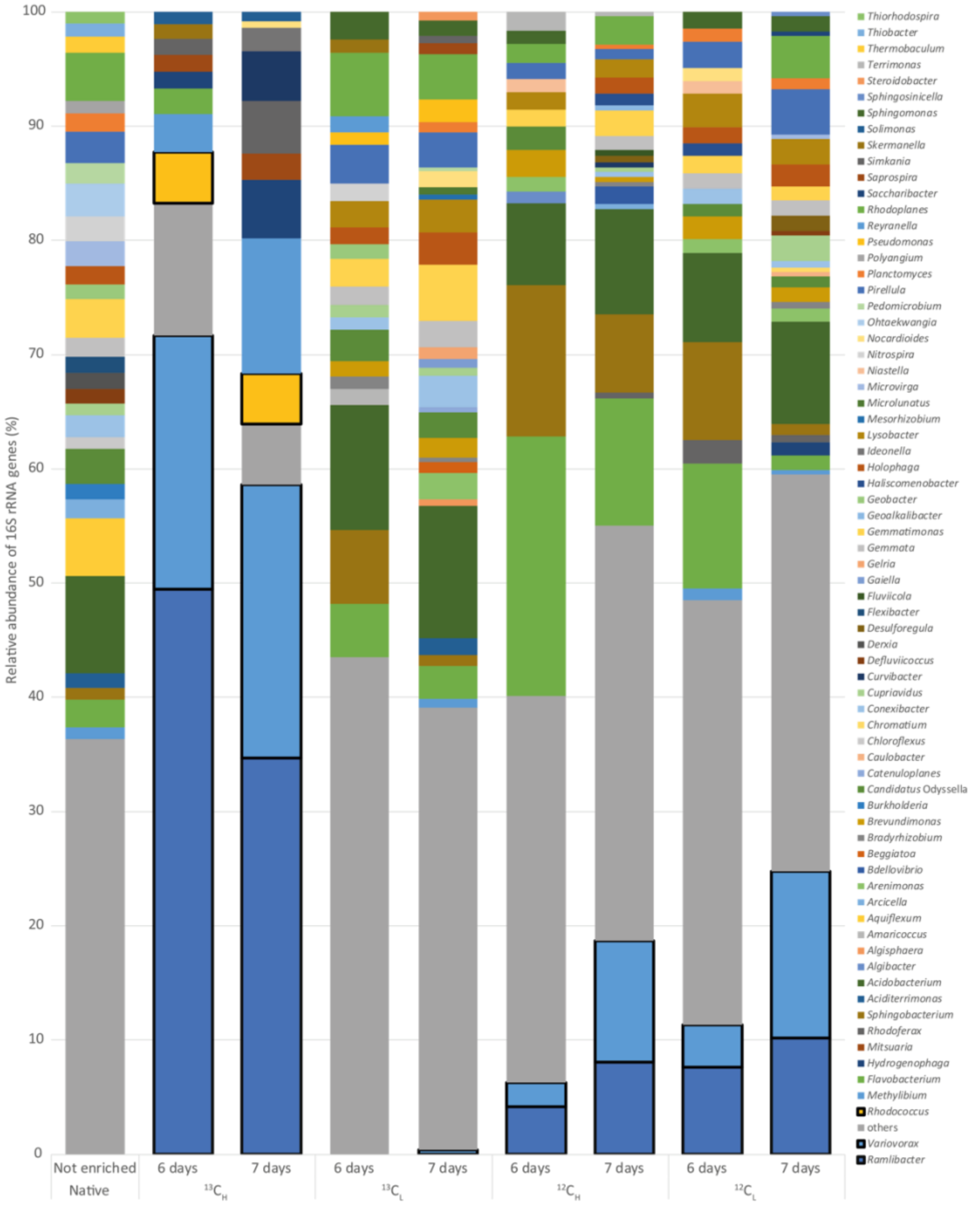

Figure S2. Relative abundance of 16S rRNA genes at the genus level in DNA isolated after DNA-SIP enrichments

The abundance of bacterial 16S rRNA genes retrieved by PCR from un-enriched (unfractionated DNA) is shown on the left. Subsequent bars describe the abundance of 16S rRNA genes retrieved from DNA-SIP experiments with isoprene-enriched willow soil samples after 6 and 7 days of enrichment with ^{12}C- and ^{13}C-labelled isoprene. DNA fractions arising after fractionation from willow soil samples enriched with ^{13}C-isoprene and ^{12}C-isoprene are designated heavy (H) and light (L), refer to Figure S5 and S6. Taxonomic affiliation of 16S rRNA genes is reported at the genus level. Only 16S rRNA gene sequences with a relative abundance of greater than 1% are shown. 16S rRNA gene sequences with a relative abundance of less than 1% are grouped together as “others”. Genera (*Ramlibacter*, *Variovorax* and *Rhodococcus*) identified in these DNA-SIP experiments as putative isoprene-degrading bacteria according to ^{13}C-labelling, are highlighted with a black border

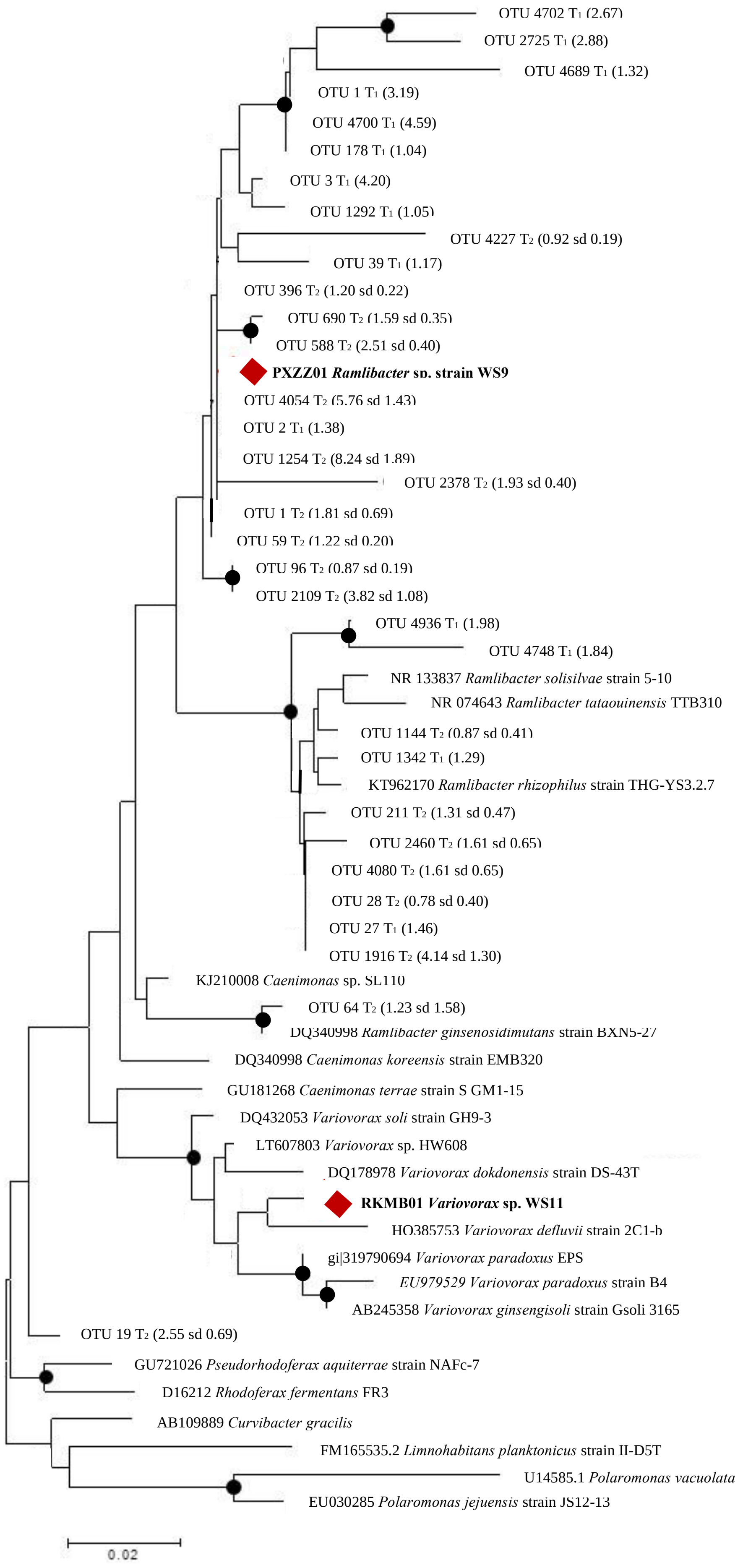

OTU 4702 T_1 (2.67)
OTU 2725 T_1 (2.88)
OTU 4689 T_1 (1.32)
OTU 1 T_1 (3.19)
OTU 4700 T_1 (4.59)
OTU 178 T_1 (1.04)
OTU 3 T_1 (4.20)
OTU 1292 T_1 (1.05)
OTU 4227 T_2 (0.92 sd 0.19)
OTU 39 T_1 (1.17)
OTU 396 T_2 (1.20 sd 0.22)
OTU 690 T_2 (1.59 sd 0.35)
OTU 588 T_2 (2.51 sd 0.40)
PXZZ01 *Ramlibacter* sp. strain WS9
OTU 4054 T_2 (5.76 sd 1.43)
OTU 2 T_1 (1.38)
OTU 1254 T_2 (8.24 sd 1.89)
OTU 2378 T_2 (1.93 sd 0.40)
OTU 1 T_2 (1.81 sd 0.69)
OTU 59 T_2 (1.22 sd 0.20)
OTU 96 T_2 (0.87 sd 0.19)
OTU 2109 T_2 (3.82 sd 1.08)
OTU 4936 T_1 (1.98)
OTU 4748 T_1 (1.84)
NR 133837 *Ramlibacter solisilvae* strain 5-10
NR 074643 *Ramlibacter tataouinensis* TTB310
OTU 1144 T_2 (0.87 sd 0.41)
OTU 1342 T_1 (1.29)
KT962170 *Ramlibacter rhizophilus* strain THG-YS3.2.7
OTU 211 T_2 (1.31 sd 0.47)
OTU 2460 T_2 (1.61 sd 0.65)
OTU 4080 T_2 (1.61 sd 0.65)
OTU 28 T_2 (0.78 sd 0.40)
OTU 27 T_1 (1.46)
OTU 1916 T_2 (4.14 sd 1.30)
KJ210008 *Caenimonas* sp. SL110
OTU 64 T_2 (1.23 sd 1.58)
DQ340998 *Ramlibacter ginsenosidimutans* strain BXN5-27
DQ340998 *Caenimonas koreensis* strain EMB320
GU181268 *Caenimonas terrae* strain S GM1-15
DQ432053 *Variovorax soli* strain GH9-3
LT607803 *Variovorax* sp. HW608
DQ178978 *Variovorax dokdonensis* strain DS-43T
RKMB01 *Variovorax* sp. WS11
HO385753 *Variovorax defluvii* strain 2C1-b
gi|319790694 *Variovorax paradoxus* EPS
EU979529 Variovorax paradoxus strain B4
AB245358 *Variovorax ginsengisoli* strain Gsoli 3165
OTU 19 T_2 (2.55 sd 0.69)
GU721026 *Pseudorhodoferax aquiterrae* strain NAFc-7
D16212 *Rhodoferax fermentans* FR3
AB109889 *Curvibacter gracilis*
FM165535.2 *Limnohabitans planktonicus* strain II-D5T
U14585.1 *Polaromonas vacuolata*
EU030285 *Polaromonas jejuensis* strain JS12-13
0.02

Figure S3. 16S rRNA gene comparisons with newly isolated isoprene-degraders and 16S rRNA genes retrieved in DNA-SIP experiments.

Neighbour-joining phylogenetic tree with partial 16S rRNA gene sequences from representative members of the Comamonadaceae family, strains isolated in this study and the most abundant 16S rRNA gene sequence OTUs (>1% abundance) obtained from the willow soil DNA-SIP experiment. OTU sequences are differentiated between sequences obtained after 6 days (T_1) and 7 days of enrichment (T_2). OTU designations, for timepoint 2, include the average relative abundance (%) and standard deviation values in parentheses. 55 16S rRNA gene sequences were included in the analysis, with a total of 251 nucleotides used in the final dataset. Bootstrap values (1000 replications,[56]) over 75% are shown as solid circles in the nodes. Isoprene-degrading strains isolated in this study are indicated in bold and with a red diamond. The scale bar shows nucleotide substitutions per site.

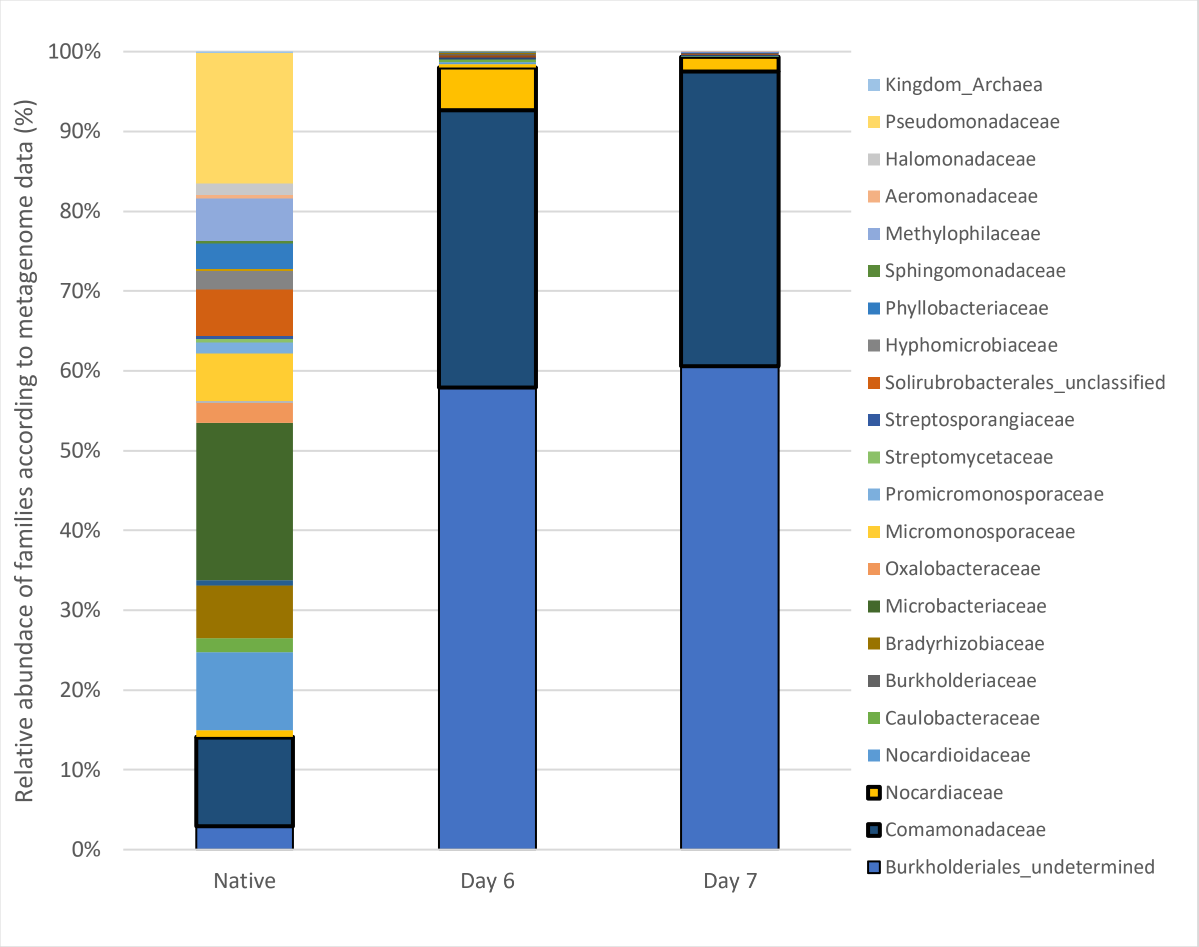

Figure S4. Metagenome analysis of ^{13}C-DNA at the family level obtained after DNA-SIP experiments.

Phylogenetic abundance obtained from un-enriched (unfractionated DNA) and ^{13}C-enriched DNA obtained from willow soil samples in DNA-SIP experiments after 6 and 7 days of enrichment with ^{13}C-labelled isoprene. Analysis was done using Metaphlan software using trimmed unassembled metagenome reads and the taxonomic affiliation is shown at the family level. Sequences identified as from putative isoprene-degrading bacteria, (according to ^{13}C-labelling) are indicated with a black border

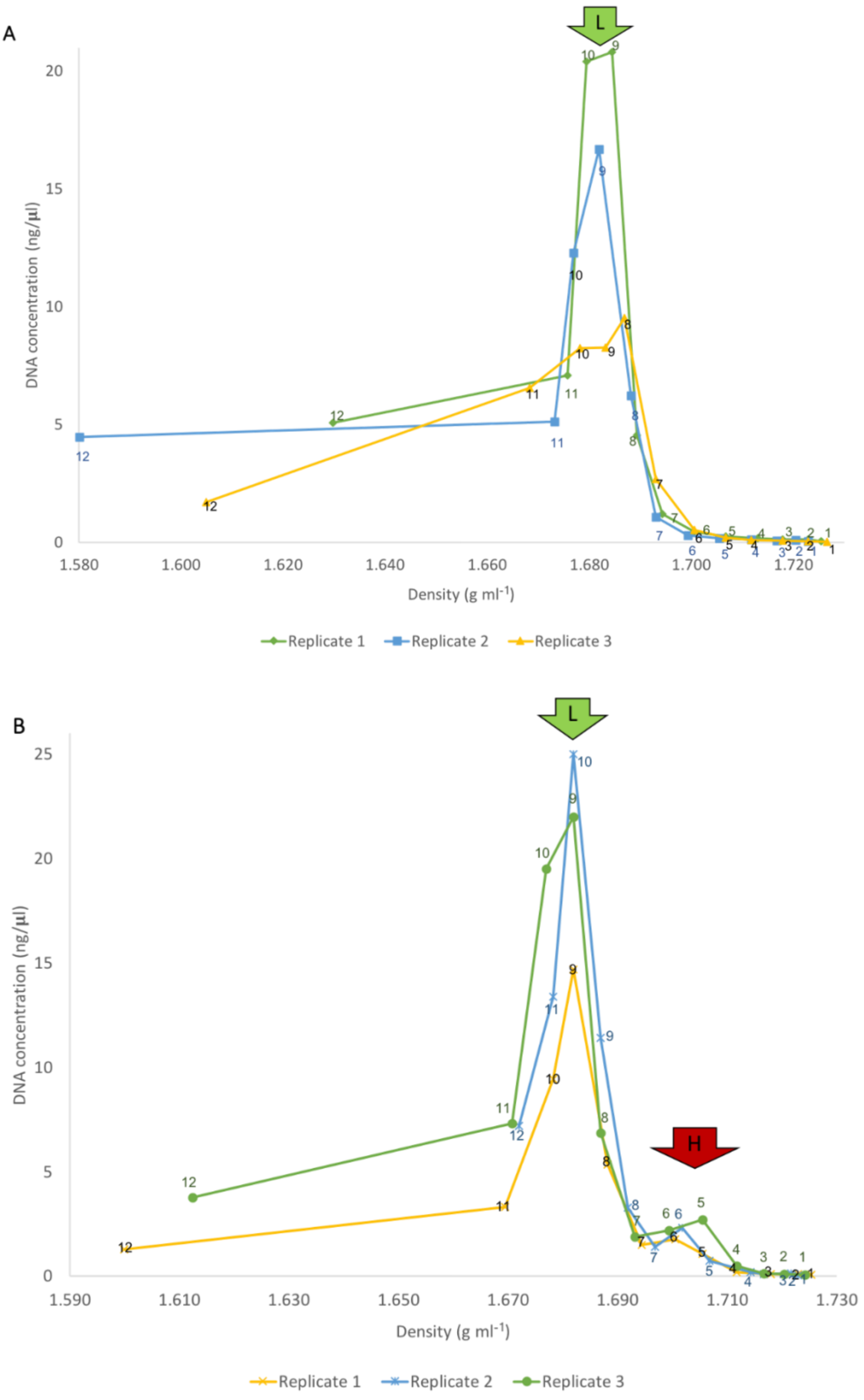

Figure S5. Separation of DNA after DNA-SIP experiments (6-day incubation).
DNA concentration and density of CsCl fractions obtained after ultracentrifugation of extracted DNA from enriched microcosms after 6 days of enrichment with A) ^{12}C-isoprene and B) ^{13}C-labelled isoprene. The CsCl fractions chosen to represent ^{13}C-labelled (heavy fraction) and unlabelled ^{12}C-DNA (light fraction) are indicated: H arrow: heavy DNA; L arrow: light DNA.

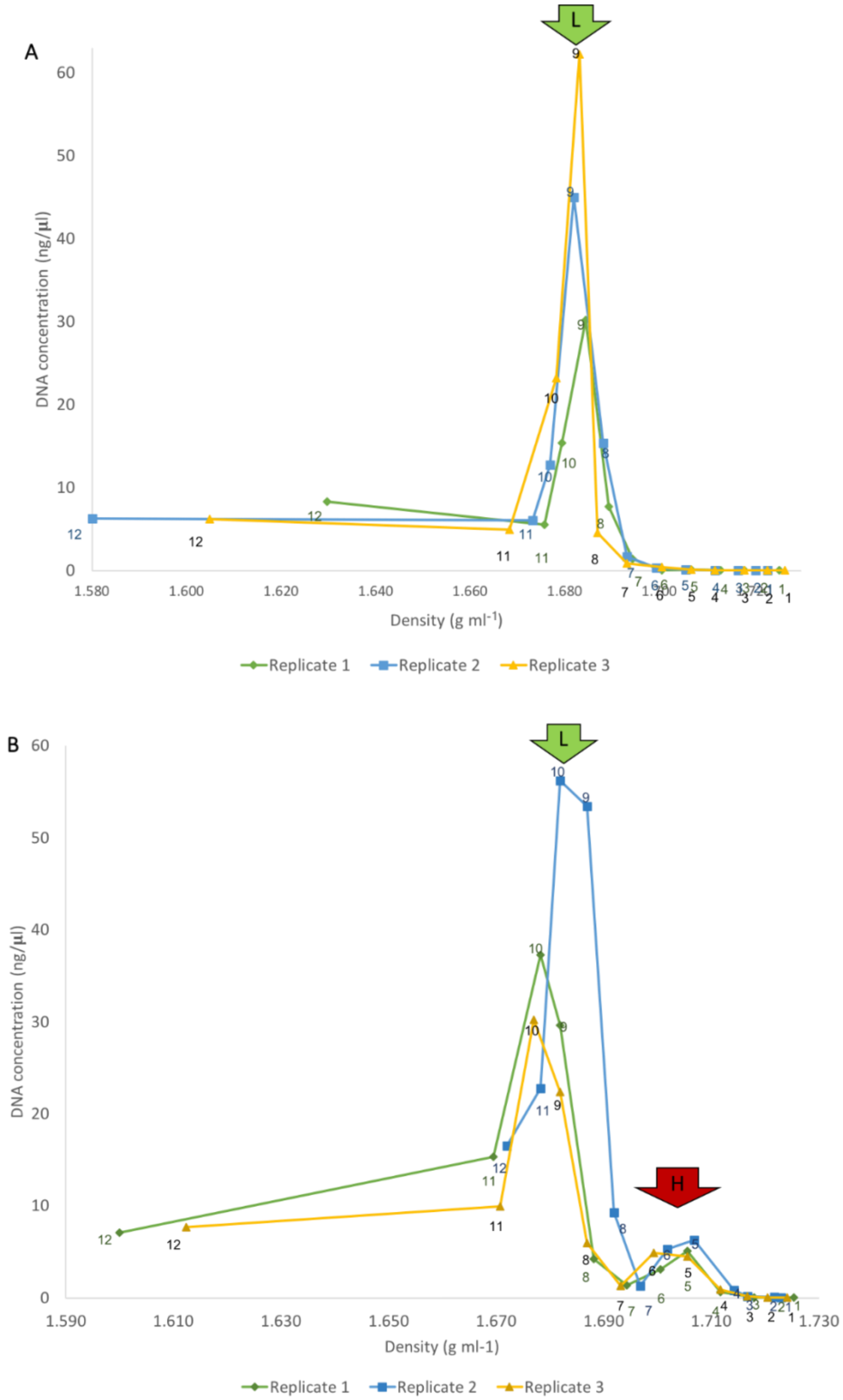

Figure S6. Separation of DNA after DNA-SIP experiments (7-day incubation).
DNA concentration and density of CsCl fractions obtained after ultracentrifugation of extracted DNA from enriched microcosms after 7 days of enrichment with A) ^{12}C-isoprene and B) ^{13}C-labelled isoprene. The CsCl fractions chosen to represent ^{13}C-labelled (heavy fraction) and unlabelled ^{12}C-DNA (light fraction) are indicated: H arrow: heavy DNA; L arrow: light DNA

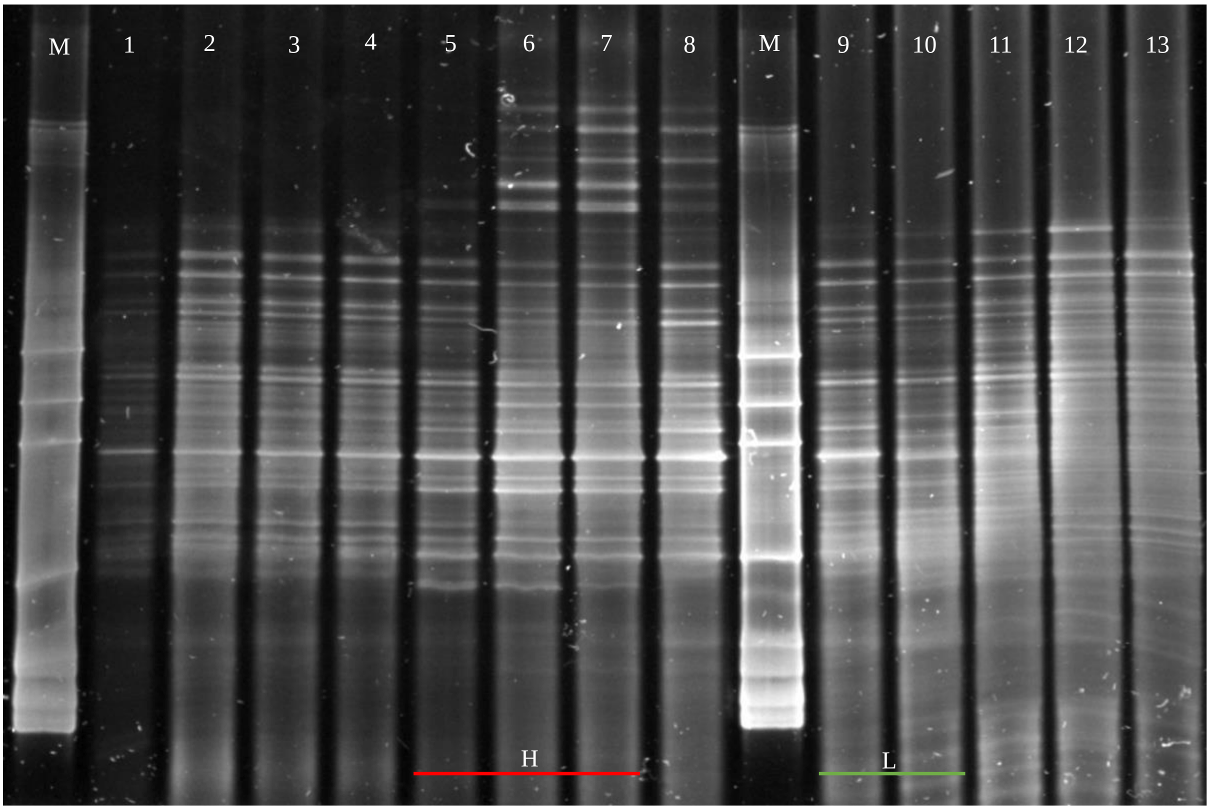

Figure S7.
Representative denaturing gradient gel electrophoresis (DGGE) gel of CsCl fractions 1 to 13 after ultracentrifugation of extracted DNA from enriched microcosms after 6 days incubation with ^{13}C-labelled isoprene. Each fraction number is shown on the top, along with the DGGE marker ladder (M). Samples that were considered light (L) and heavy (H) are shown with a green and red line below, respectively.

Reference:

Crombie, A. T., Khawand, M. El, Rhodius, V. A., Fengler, K. A., Miller, M. C., Whited, G. M., et al. (2015). Regulation of plasmid-encoded isoprene metabolism in Rhodococcus, a representative of an important link in the global isoprene cycle. *Environ. Microbiol.* 17, 3314–3329. doi:10.1111/1462-2920.12793.

Rodriguez-R, L. M., Gunturu, S., Harvey, W. T., Rosselló-Mora, R., Tiedje, J. M., Cole, J. R., et al. (2018). The Microbial Genomes Atlas (MiGA) webserver: taxonomic and gene diversity analysis of Archaea and Bacteria at the whole genome level. *Nucleic Acids Res.* 46, W282–W288. doi:10.1093/nar/gky467.
